# An Effective Expanded Graphite Coating on Polystyrene Bead for Improving Flame Retardancy

**DOI:** 10.3390/ma14216729

**Published:** 2021-11-08

**Authors:** Minjung Bae, Hyunhwa Lee, Gyeongseok Choi, Jaesik Kang

**Affiliations:** 1 Department of Building Energy Research, Korea Institute of Civil Engineering and Building Technology, Goyang 10223, Korea; baeminjung@kict.re.kr (M.B.); hhlee@kict.re.kr (H.L.); bear717@kict.re.kr (G.C.); 2 Department of Smart Convergence Architecture, College of Engineering, Ajou University, Suwon 16499, Korea

**Keywords:** fire safety standards, expanded polystyrene, expanded graphite, semi-nonflammable

## Abstract

Although foamed plastic insulation is widely used in construction in the Korean market, it is vulnerable to fire. To improve the flame retardancy, the method of flame-retardant coating with the EG in water-soluble state on the surface of expanded polystyrene (EPS) beads has been widely used. However, polystyrene beads coated with a water-soluble flame retardant easily separate the coated flame retardant in manufacturing. In this study is devised a flame-retardant coating and two steps of coating process for adhering the flame-retardant coating film evenly to the surface of the polystyrene bead without exfoliation. It was analyzed whether a flame-retardant EPS (FR-EPS) with excellent flame retardancy could be manufactured using polystyrene beads coated in this way. Ten FR-EPS samples satisfied the HF-1 and V-0 levels in horizontal and vertical burning tests, respectively. The THR of eight FR-EPS samples for ten minutes did not exceed 8 MJ∙m^−2^ and the maximum HRR did not exceed 200 kW∙m^−2^ for more than ten consecutive seconds. FR-EPS passed the building material standard of semi-nonflammability in Korean regulations, in contrast to commercial EPS, which have not passed the semi-nonflammability standard. It was also analyzed how effective the designed coating is in this study, comparing it with composites that were planned to improve the flame resistance of polystyrene, as reported in the literature. Flame Retardancy Index (FRI) values of FR-EPS proved the “excellent” level and had higher values compared with other polystyrene composites. These results demonstrated that the coated EPS containing a water-soluble flame retardant manufactured from EG and two steps of application with the coating solution achieved fire safety standard regulations.

## 1. Introduction

The Korean government has begun to implement policies to strengthen the flame retardancy of building insulation. Korean regulations [1] provide test methods and standards for classifying building finishes, including insulation, as nonflammable, semi-nonflammable, and flame-retardant materials. The external insulation system should satisfy the regulation [2] for thermal performance on each part of the building and include a fire prevention structure defined by the regulation or consist of building materials certified for the performance of being semi-nonflammable. The reinforced policy calls for building insulation products that secure fire safety and thermal performance at the same time.

Foamed plastic insulations, especially expanded polystyrene (EPS), are widely used as insulation materials for buildings in Korea due to their inexpensive price, sufficient thermal performance, and easy construction. However, when exposed to fire, it easily ignites and emits a large amount of heat and smoke as it burns, making it relatively vulnerable to fire [3,4,5].

To improve the flame retardancy of foam plastic insulations, a flame retardant is added to the insulation, or flame-retardant components, such as phosphorus and nitrogen, are chemically combined to use a raw material. Flame retardants can also be classified as reactive and additive. A reactive type of flame retardant is chemically bonded to the side chain to impart flame retardancy. It introduces a monomer capable of imparting flame retardancy to the main chain of the polymer to prepare a flame-retardant polymer, or introduces a reactive group into the polymer to form the end of the polymer. An additive type of flame retardant is added as an additive during the compounding process and is simply mixed. Currently, an additive type of flame retardant is commonly used to improve the flame retardancy of foam plastic insulation [6,7]. The additive type of flame retardant has low cost and easily improves the flame retardancy of polymer materials, as compared with the reactive type of flame retardant [8]. To add a flame retardant into foam plastic insulation, it should have good mixing properties with raw materials and additives, should not affect the physical characterization of the final products, and should generate low amounts of smoke and toxic gas during combustion [9].

Halogen elements generate gases harmful to the human body during combustion and are limited in use because gases from halogen elements corrode metals [10]. Environmentally friendly flame retardants are receiving attention to overcome the problem of halogen-based flame retardants and to comply with strict flame retardant regulations [11]. Moreover, the flame retardancy of halogen-based flame retardants weakens over time. To solve these problems, various studies have been conducted to develop a highly functional way to improve flame retardancy, such us encapsulating a flame retardant in polyurethane [12], modifying and adding an inorganic flame retardant [13], adding a functional group with flame retardancy and heat resistance to the polyurethane chain [14,15], using an organic and inorganic hybrid [16], adding expanded graphite [17], and adding nano-clay [18]. In this study, flame retardant additives based on expanded graphite (EG) were added to expanded polystyrene (EPS), and their effect was analyzed. The EG has been considered as an ideal candidate to be a halogen-free fire retardant [19]. When heat is applied after bonding a sulfur or nitrogen compound between the interlayer structures, the particles expand hundreds of times, resulting in layer separation like an accordion, and this porous structure can easily form a composite material. Chun et al. reported that the THR decreased as the amount of expanded graphite was increased from 0 to 30 g, and the sample thickness was increased up to 14 times after the combustion reaction [20]. Laachachi et al. reported that the peak Heat Release Rate (peak HRR) and the Total Heat Release rate (THR) decreased with increasing content of expanded graphite from 5 to 50 wt% in the epoxy resin material [21]. Lee et al. reported that with increasing content of expanded graphite, the THR decreased by up to 83%, and the limiting oxygen index increased by up to 44%; thus, the flame retardancy of foam materials improved [22]. Vahabi et al. reported the synergistic potential with magnesium hydroxide, expandable graphite, and expanded graphite in order to control thermal conductivity and, therefore, fire properties. It was found that there is a best composition, which is 30 wt% of magnesium hydroxide, 10 wt% of expanded graphite, and 10 wt% of expandable graphite for a total filler content of 50 wt% [23].

Since EPS has low flame-retardant efficiency, a large amount of halogen-free flame retardant should be added during the polymerization or impregnation. It reduces the physical properties and intrinsic quality of a foamed product. For this circumstance, it is a good choice with little effect on the foaming process of coating with the EG in water-soluble state on the surface of polystyrene beads [24]. The flame-retardant coating has been widely used for EPS [25], and thermosetting resins are commonly used as adhesives in this coating technique due to their inherent fire resistance and good adhesion [24]. However, polystyrene beads coated with a water-soluble flame retardant easily separate the coated flame retardant during drying and molding process in manufacturing. This phenomenon causes the following problems; If the flame-retardant coating on the polystyrene beads is separated during the manufacturing process, the coating residue sticks to the molding machine, which decrease molding workability. Polystyrene beads from which the flame-retardant coating is peeled off reduce the thermal conductivity of product, and it is difficult to predict the effect of improving flame retardancy on the product.

In this study is devised a strategy of flame-retardant coating film adhered evenly to the surface of polystyrene beads without exfoliation. A flame-retardant coating solution was prepared using a water-soluble flame retardant manufactured from EG, a porous particle, a starch, and a water-based binder, and the coating solution was applied in two steps. It was analyzed whether the flame-retardant EPS (FR-EPS) with excellent flame retardancy could be manufactured using polystyrene beads coated in this way. Specimens of foamed plastic insulation materials that are commonly used in Korea were collected and tested to analyze their flammability level, and FR-EPS was also included in specimens. FR-EPS should pass the standard of semi-nonflammability in the regulation in order to apply it in external insulation systems in Korea. Their properties of self-extinguishing and flame retardancy were analyzed and compared with those of commercial products. The coating effect on the polystyrene bead devised in this study was analyzed using the Flame Retardancy Index (FRI) defined by Vahabi et al. [26], as a simple yet universal dimensionless criterion born out of the cone calorimeter test result, which is peak HRR, THR, and Time-To-Ignition (TTI). It has a purpose for quantifying the flame retardancy of different polymer composites on a set of reliable data. In the previous study, the improving systems of the flame retardancy for the polystyrene were analyzed to get a set of reliable data, and the dataset including the case of FR-EPS was used to calculate the FRI.

## 2. Materials and Methods

### 2.1. Description of FR-EPS

The EPS foam manufacturing process is divided into two steps: preparing expandable polystyrene beads and fabrication of the beads into a finished cellular plastic article [27]. FR-EPS was applied with a flame retardant manufactured from EG supplied by Nabotec Co., Ltd. EG has various nominal particle sizes (70~960 μm) and loads, and if EG has a large dimension, it leads to poor dispersion and compatibility in adhesives. The small dimension may be considered in order for the graphite-based material to be uniformly dispersed; however, as the particle size of EG decreases, the expanded volume and thickness decrease, which will lead to the weakening of flame retardant performance [28]. This study used the EG that the average particle sizes was 450 μm.

The flame-retardant coating solution contains 10 to 30 wt% of a porous particle, 5 to 20 wt% of a starch, 20 to 50 wt% of a flame retardant manufactured with the EG, and 20 to 30 wt% of a water-based binder. A total of 0.1 to 3 wt% of a silane (silicone compound) and 3 to 15 wt% of a thermosetting resin are included as additives. The solution should be coated on the polystyrene bead surface. The water-based binder serves as an adhesive to allow the flame-retardant solution to adhere to the bead surface. However, the water-based binder has a flammable property, the amount of the water-based binder needs to be reduced by adding the starch. The water-based binder contains 50 to 65 wt% of water as a solvent, and 35 to 50 wt% of a water-soluble polyvinyl acetate resin. The flame retardant manufactured with the EG is preferably included in an amount of 20 to 50 wt% in the flame retardant coating solution. If the flame retardant is less than 20 wt%, the flame retardancy is poor, and if it is 50 wt% or more, the foamability is lowered when foaming polystyrene beads as well as bad adhesion between beads.

**Figure 1:**
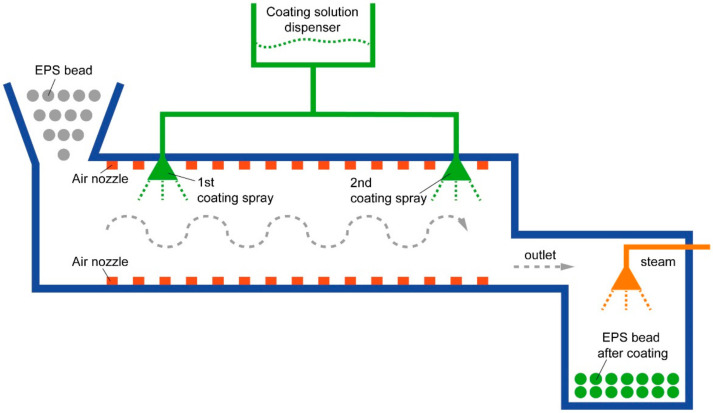
Case for coating polystyrene beads with the flame-retardant coating solution.

For coating with the flame-retardant solution, polystyrene beads enter in the case that creates a vortex inside, as shown in Figure 1. All beads rotate inside the case and move to the outlet. At this time, the first coating spray operates to coat the polystyrene beads with the flame-retardant coating solution, when beads move to under the second coating spray outlet by the vortex, the spray operates for re-coating with the solution. These sprays coat the surface of polystyrene beads several times at a distance of time. It is possible to prevent the flame-retardant solution from being coated on the surface, and it can be coated evenly. 

**Figure 2:**
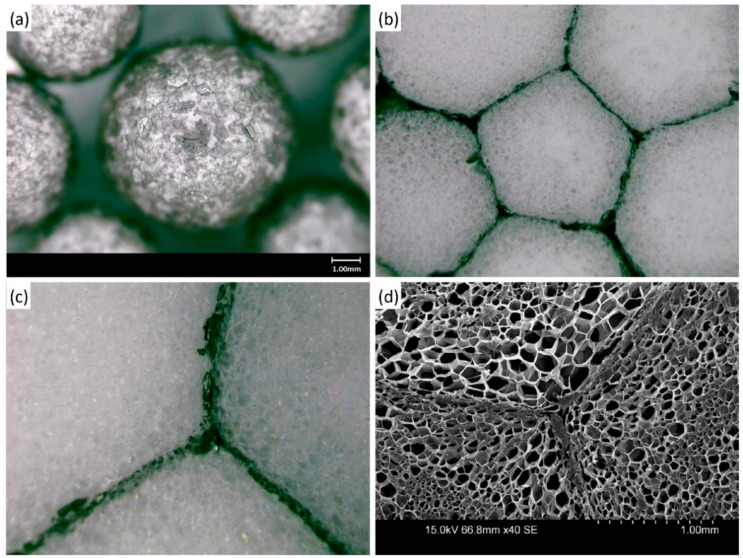
(**a**) Bead coated using a water-soluble flame retardant manufactured from expanded graphite, (**b**) Surface of FR-EPS using the beads, (**c**) Magnified image between beads of the FR-EPS surface, (**d**) (SEM) images of the FR-EPS surface.

Figure 2a shows the polystyrene bead coated with the flame-retardant solution. It is expected to improve the flame retardancy of the product because the uniformly coated beads are used in manufacturing. When steam is shot on the beads, these are foamed and melted to fuse the beads with each other. In this way, the foam is completed with FR-EPS, and it is cut and manufactured into an insulation panel. Figure 2b shows the surface of FR-EPS, which is foamed using the uniformly coated polystyrene beads. Figure 2c presents magnified image between beads of the FR-EPS surface, and Figure 2d presents scanning electron microscopy (SEM) images of the FR-EPS surface. There is no collapse of the polystyrene cells, and a water-soluble flame retardant penetrated into cells on the bead surface, forming a flame-retardant film between the bead. The FR-EPS was tested if it could be defined as a KS product. The test results satisfied the EPS criteria of Type 1 and No. 3 specified in KS M 3808 [29]: The density is 20 kg∙m^−3^ or more, the initial thermal conductivity at average temperature (23 ± 2) °C is 0.040 W∙m^−1^∙K^−1^ or less, the bending load is higher than 25 N, and the compressive stress is higher than 8 N.

### 2.2. Sample Preparation

Korean standard (KS) evaluates the combustion characteristics only in the horizontal direction, but the combustion characteristics should be evaluated in the vertical direction as well when a fire occurs in a building. First, the horizontal burning test followed KS M ISO 9772 [30], and the vertical burning test followed UL 94 [31] by Underwriters Laboratories. For these tests, twenty-five of Type 1 and thirty-four of Type 2 EPS products certified by KS M 3808 and nine extruded polystyrene (XPS) insulation types certified by KS M 3808 were randomly sampled as specimens. The specimens contained ten FR-EPS samples. Second, the flame retardancy of foamed plastic insulations commercially available in Korea was analyzed according to the cone calorimeter method, which followed KS F ISO 5660-1 [32]. For this analysis, two samples of EPS-Type 1, two samples of EPS-Type 2, one sample of XPS, one sample of polyisocyanurate (PIR) type 1 and polyurethane (PUR) foam, and one sample of phenolic foam (PF) were randomly sampled as specimens among the building insulations certified by KSs. In addition, eight FR-EPS samples were also tested according to the cone calorimeter method.

### 2.3. Testing Methodology

#### 2.3.1. Burning Tests in the Horizontal and Vertical Directions

The burning test method according to KS M ISO 9772 [30] requires a test specimen with a thickness of 13 mm, length of 150 mm, and width of 50 mm. The method presents a classification system that is used to characterize the burning behavior of cellular plastic materials with densities less than 250 kg∙m^−3^. The class is determined by examining the test results for specimens, and each class represents a range of performance levels that simplify descriptions in specifications. One class that best matched the material performance according to the requirements of Table 1 was chosen. The distance burnt is the distance between a 25 mm gauge mark and the point where the flame or glowing combustion stopped was expressed in millimeters. If the flame front went out before the 25 mm mark, 0 mm was recorded. The burning time was the time measured by the second timing device in seconds, from when the flame or glowing combustion passed the 25 mm gauge mark until the flame front stopped or passed a 125 mm gauge mark. In the order of HF-1 > HF-2 > HBF, the self-extinguishing behavior in the horizontal direction was determined to be excellent.

UL 94, established by Underwriters Laboratories, describes the burning test procedures in the vertical and horizontal directions. In this study, a 50 W vertical burning test method using a 20 mm flame was chosen to analyze the burning characteristics of cellular plastic materials. It defines the combustible properties when standard samples react to a small flame or radiant heat source under controlled laboratory conditions. The specimen was 125 mm long and 13 mm wide and had a minimum thickness of 3 mm. The maximum thickness did not exceed 13 mm. The specimen was clamped from the upper 6 mm of the specimen, along the longitudinal axis oriented vertically, so that the lower end of the specimen was 300 mm above the horizontal layer. The flame was applied centrally to the middle point of the bottom edge of the specimen so that the top of the burner was 10 mm below the point of the lower end of the specimen. The specimen contacted the burner twice perpendicularly for 10 s, and the total combustion, including the indicator ignition, was examined, and the material was classified, as shown in Table 2. A set of five specimens was required for the test, and the average value of five test specimens was used for class determination. It was concluded that the self-extinguishing property in the vertical direction was excellent in the order of V-0 > V-1 > V-2.

#### 2.3.2. Reaction-to-Fire Test of the Total Heat Release Rate (Cone Calorimeter Method)

In Korea, enforcement rules for building fire protection structures stipulate technical standards for building evacuation and fire protection. Results of testing according to the KSs should satisfy the performance standards of flame-retardant materials established by the Korean government. The Korean code for the flame retardant performance of building finishing materials and the fire prevention structure specifies a classification system for flame-retardant materials. The class is defined as nonflammable, semi-nonflammable, and flame retardant and determined based on the test results according to standard methods. The test for gas toxicity should follow KS F 2271 [33], and in all classes, the average inactive time of laboratory rats should exceed 9 min. For the class of semi-nonflammable materials, test results should meet KS F ISO 5660-1. In particular, the THR for 10 min after the start of a heating test should be 8 MJ∙m^−2^ or less, and the maximum heat release rate for 10 min should not exceed 200 kW∙m^−2^ for more than 10 consecutive seconds. In addition, after heating for 10 min, there should be no cracks, holes, or melting (including the melting and disappearance of all the inner material in the case of a composite material) that penetrates the specimen for fire protection. To belong to the class of flame-retardant materials, a specimen tested by the same method should meet the following requirements: The THR for 5 min after the start of a heating test should be 8 MJ∙m^−2^ or less, and the maximum heat release rate for 5 min should not exceed 200 kW∙m^−2^ for more than 10 consecutive seconds. In addition, after heating for 5 min, there should be no cracks, holes, or melting (including the melting and disappearance of all the inner material in the case of a composite material) that penetrates the specimen for fire protection.

**Figure 3:**
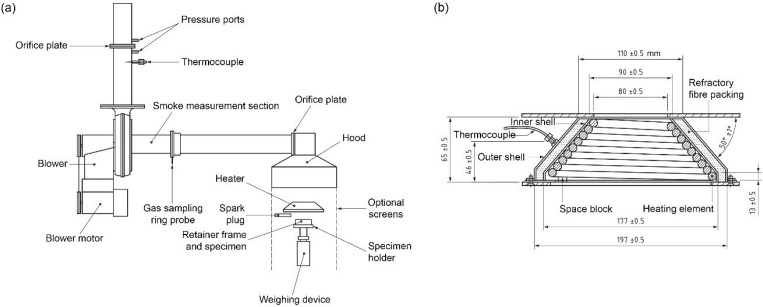
Test apparatus according to KS F ISO 5660-1 (**a**) Apparatus (**b**) Cone heater.

In this study, specimens of the foam plastic insulation material were tested according to KS F ISO 5660-1 to analyze the minimum level of fire safety required for a component of an external building insulation system. This method was used to assess the heat release rate and dynamic smoke production rate of specimens exposed in the horizontal orientation to controlled levels of irradiance with an external igniter. As shown in Figure 3, the active element of the heater consisted of an electrical heater rod, capable of delivering 5000 W at the operating voltage, tightly wound into the shape of a truncated cone. This is called the cone calorimeter method. The test method is based on the observation that the net heat of combustion is generally proportional to the amount of oxygen required for combustion. The relationship is that approximately 13.1 MJ of heat is released per kilogram of oxygen consumed. The specimens in the test were burned under ambient air conditions, while being subjected to a predetermined external irradiance within the range of 0 to 75 kW∙m^−2^. The oxygen concentrations and the exhaust gas flow rates were measured. This test method was used to assess the contribution of the product under test to the rate of heat evolution during its involvement in fire.

## 3. Results and Discussion

### 3.1. Burning Test Results in the Horizontal and Vertical Directions

Table 3 shows the results of the horizontal and vertical burning tests of nine samples of XPS, twenty-five samples of EPS Type 1, and thirty-four samples of EPS Type 2. First, a number from one to nine was randomly assigned to the XPS samples, and it was classified like “XPS-1”. In the case of EPS samples, a random number was also assigned to each sample, and 1 or 2 was assigned between “EPS” and a random number to distinguish EPS Type 1 and 2; “EPS-1-1”.

In the horizontal burning test for the XPS samples, XPS-1 and XPS-6 did not self-extinguish, three samples, XPS-2, XPS-7, and XPS-9, satisfied the HF-1 level, and four samples, XPS-3, XPS-4, XPS-5, and XPS-8, satisfied the HF-2 level. In addition, XPS-6 did not satisfy the performance standards in the vertical burning test, and all other samples satisfied the V-2 level. Even if the HF-1 level was met, which was defined as high self-extinguishing in KS M ISO 9772, the result of the vertical burning test corresponded to a low level (V-2), and the result of the vertical burning test was the same, even though the performance level according to the horizontal burning test was different.

EPS-1-2, which is an EPS Type 1 sample, did not self-extinguish. All EPS Type 1 samples satisfied the HF-2 level except for EPS-1-25, which satisfied the HF-1 level. However, EPS-1-2 did not satisfy all performance standards in the vertical burning test, and all other samples of EPS Type 1 passed the V-2 level. In the results of the horizontal burning test of thirty-four EPS Type 2 samples, EPS-2-24 and EPS-2-31 did not self-extinguish, fourteen samples of EPS Type 2 satisfied the HF-1 level, and the remaining eighteen samples met the HF-2 level. In the vertical burning test results, EPS-2-18 and EPS-2-19 satisfied the high level (V-0) specified in UL 94, and all other samples satisfied the V-2 level. That is, in the case of EPS-2-18 and EPS-2-19, a high level of self-extinguishing was verified according to the horizontal and vertical burning tests. However, a test result with excellent self-extinguishing, such as the HF-1 level in the horizontal burning test, did not lead to high self-extinguishing, such as V-0 in the vertical burning test. Likewise, the results of the horizontal burning test could not be inferred based on the results of the vertical burning test. Both horizontal and vertical burning tests were required to evaluate the self-extinguishing property (combustibility) of the foam polystyrene insulation samples.

Table 4 shows the test results for ten samples of FR-EPS. As can be observed, fire safety was improved by using flame-retardant additives manufactured from expanded graphite. All FR-EPS samples satisfied the HF-1 level in the horizontal burning test. In addition, in the vertical burning test, all samples were analyzed to satisfy the V-0 level; therefore, self-extinguishing in the horizontal and vertical directions was improved by one and two steps, respectively, as compared with commercial EPS.

### 3.2. Results of the Reaction-to-Fire Test of the Total Heat Release Rate (Cone Calorimeter Method)

**Figure 4:**
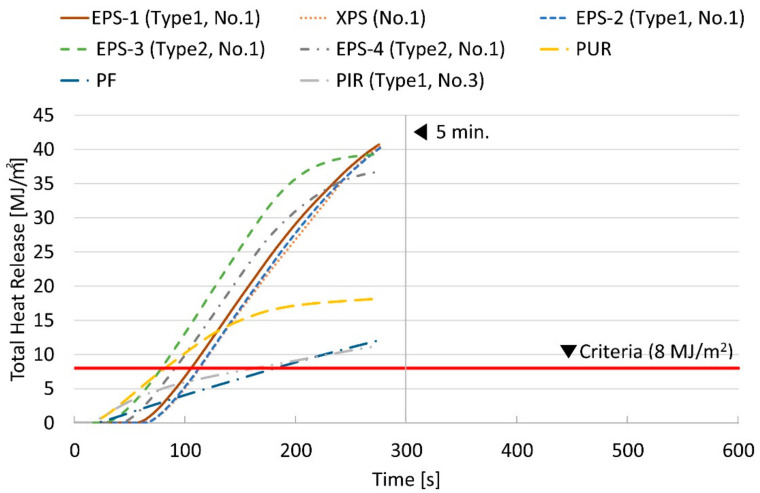
THR with foamed plastic insulation specimens according to the cone calorimeter.

Figure 4 shows the THRs for eight samples of foamed plastic insulation. All samples did not pass the standard for flame retardant materials in the regulation because their THR exceeded 8 MJ∙m^−2^ before 3 min from the start of the test. The test was terminated 275 s after the start of the test.

**Figure 5:**
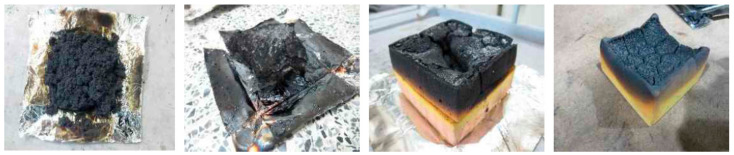
The specimen after 5 min of conducting the cone calorimeter method.

**Figure 6:**
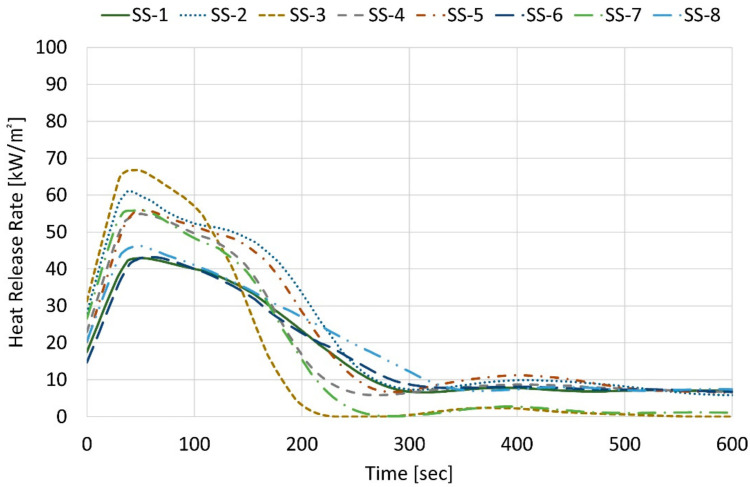
HRR of flame-retardant EPS samples according to the cone calorimeter method.

The four samples of EPS showed the THR between 36.7 and 40.7 MJ∙m^−2^, and one XPS sample showed 40.5 MJ∙m^−2^. In Figure 4, their THRs of samples, EPS-1, XPS, EPS-2~4 exceed 8 MJ∙m^−2^ before 2 min after starting the test. PUR, PF, and PIR samples did not pass the minimum level of flame-retardant materials indicated in the regulation because their THR exceeded 8 MJ∙m^−2^ before 4 min. In addition, all samples had cracks, holes, and melting throughout the specimens, as shown in Figure 5, when the test was completed according to the cone calorimeter method. Table 5 shows the cone calorimeter test results for eight FR-EPS samples; SS-1 to SS-8, and Figure 6 shows the HRR for each sample.

**Figure 7:**
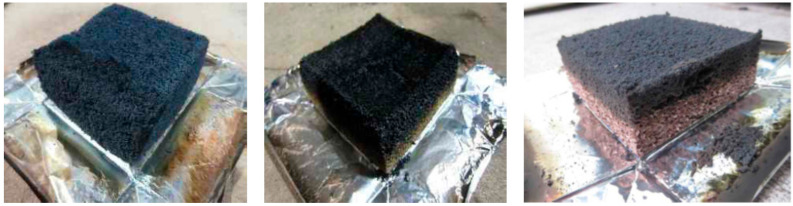
FR-EPS samples after the cone calorimeter test.

The HRR refers to the instantaneous amount of heat generated per surface area and can be used as an index to analyze the risk of an initial fire. The lower value of the peak HRR and THR, the higher the fire resistance of composites [34]. The HRR of the FR-EPS samples increased rapidly within 50~60 s, but the maximum HRR was found to be 43~67 kW∙m^−2^. The THR of all FR-EPS samples did not exceed 8 MJ∙m^−2^ at 5 min after starting the test. SS-1 and SS-8 were burned for 5 min, and no combustion phenomena occurred because of the carbonization effect. After starting the test, the FR-EPS samples passed the standard for semi-nonflammable materials in the regulation because the THR during 10 min did not exceed 8 MJ∙m^−2^ and because the maximum HRR for 10 min did not exceed 200 kW∙m^−2^ for more than 10 consecutive seconds. As shown in Figure 7, after burning for 10 min, there were no cracks, holes, or melting throughout the FR-EPS specimens.

It was analyzed how effective the designed coating was compared with the composites, which were planned to improve the flame resistance of the polystyrene, as according to the literature. Table 6 shows the extracted data from the literatures on cone calorimeter test result of the polystyrene composites. Even though there were lots of studies about the improved flame retardancy of the polystyrene, the limited literature could be used for the calculation of FRI because three parameters, peak HRR, THR, and TTI, were rarely reported in the same study. Among the literature was compared using of EG as a flame retardant or coating of a flame retardant on polystyrene beads.

The FRI was defined as the below equation:

(1) $$\mathrm{Flame}\;\mathrm{Retardancy}\;\mathrm{Index}\;\left(\mathrm{FRI}\right)\;=\;\frac{{\left[\mathrm{THR}*\frac{\mathrm{peak}\;\mathrm{HRR}}{\mathrm{TTI}}\right]}_{\mathrm{Neat}\;\mathrm{polymer}}}{{\left[\mathrm{THR}*\frac{\mathrm{peak}\;\mathrm{HRR}}{\mathrm{TTI}}\right]}_{\mathrm{Composite}}}$$

**Figure 8:**
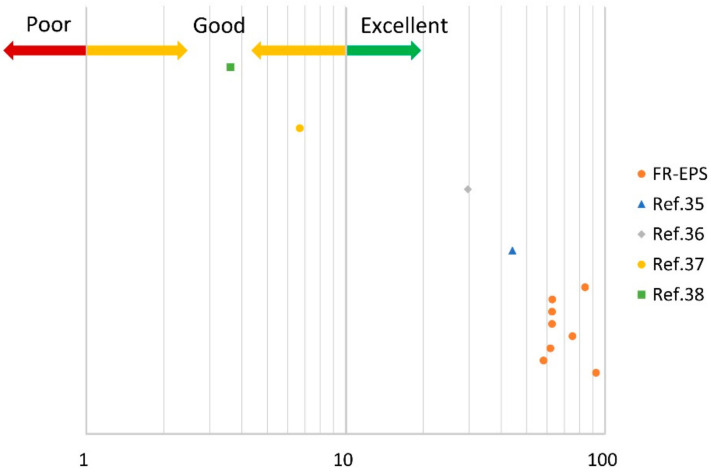
The calculated FRI for FR-EPS and the polystyrene composites containing the flame retardant.

It defined a ratio of between the neat polymer and the composite containing only one flame retardant additive because it supports determining the influence of filters or additives on the flame retardant behavior and properties [26]. To compare the flame retardancy of the composites selected from the previous literature through various types of additives or fillers, even if more than one flame retardant material was used, it was used in the calculation of the FRI, and the result is shown in Figure 8.

FRI < 1 symbolized “poor”, taken the lowest level of flame resistance. This meant that applying a flame retardant did not help improve the flame retardancy. If the applied flame retardant is effective, the calculated FRI value might be greater than 1. Thus, 1 < FRI < 10 symbolized “good”, and the FRI value in 10 < FRI < 100 indicated “excellent”. All of [35,36,37,38] satisfied FRI > 1, and 44.1 was calculated as FRI value for [35]. The FRI values of FR-EPS satisfied 58.2~92.7, and it can be seen that FR-EPS had a more efficient flame retardant strategy than the improved polystyrene composites from the literature.

## 4. Conclusions

Foam plastic insulation, for example, EPS, is widely used in buildings because of its advantages of low cost and construction convenience. The Korean government operates various regulations for fire safety in buildings, and only insulation materials for construction can be used at the performance level defined in the regulations. In order for a building insulation to be used on the exterior wall, the self-extinguishing property of insulation should satisfy the standards defined by the regulations. In this study, flame retardant additives based on EG were added to EPS for improving the flame resistance. The way of coating flame retardant on the surface of polystyrene beads is selected, which is already widely used in EPS, but the coating is needed to improve so that it adheres evenly to the surface of polystyrene beads without peeling. The FR-EPS, which was developed by coating an additive type of flame retardant manufactured from expanded graphite on commercial EPS was analyzed to achieve the fire safety standard in regulations.

In tests of commercial products, even if an insulation sample satisfied the high self-extinguishing level in the horizontal burning test, its level was low in the vertical burning test. The same results were obtained in the vertical burning test, even though the results of the horizontal burning test were different. When the self-extinguishing behavior of the foam plastic insulation sample was evaluated, it was clear that the burning test should be conducted simultaneously in the horizontal and vertical directions. The commercial foamed plastic insulation sample did not meet the performance standards for nonflammable, semi-nonflammable, and flame-retardant materials indicated in the regulations.

The horizontal and vertical burning tests with FR-EPS confirmed a high level of self-extinguishing, HF-1, and V-0. Compared with commercial polystyrene products, fire safety in the horizontal direction improved to one level, and in the vertical direction, the fire safety improved to two levels. Through the cone calorimeter test, FR-EPS passed the building material standard of semi-nonflammability in regulations. Its THR for 10 min did not exceed 8 MJ∙m^−2^, and the maximum HRR for 10 min did not exceed 200 kW∙m^−2^ for more than 10 consecutive seconds. After burning for 10 min, there were no cracks, holes, or melting throughout the specimen. It was also analyzed how effective the designed coating in this study was, comparing it with composites planned to improve the flame resistance of the polystyrene, as reported in the literature. FRI values of FR-EPS proved the “excellent” level and had higher FRI values compared with FRI values of other polystyrene composites. These results demonstrated that the coated EPS containing a water-soluble flame retardant manufactured from EG and two steps of application with the coating solution achieved fire safety standard in regulation.

## Figures and Tables

**Table 1:** Classifications of the burning behavior according to KS M ISO 9772.

Criteria Conditions	HF-1	HF-2	HBF
Linear burning rate	N/A	N/A	40 mm/min
After-flame time for each individual specimen	4/5 ≤ 2 s 1/5 ≤ 10 s	4/5 ≤ 2 s 1/5 ≤ 10 s	N/A
After-glow time for each individual specimen	≤30 s	≤30 s	N/A
Cotton indicator ignited by flaming particles or drops	No	Yes	N/A
Damaged length for each individual specimen	≤60 mm	≤60 mm	≥60 mm
4/5: Four out of a set of five specimens 1/5: One out of a set of five specimens			

**Table 2:** Classifications of the burning behavior according to KS M ISO 9772.

Criteria Conditions	V-0	V-1	V-2
After-flame time for each individual specimen	≤10 s	≤30 s	≤30 s
Total after-flame time for any condition set	≤50 s	≤250 s	≤250 s
After-flame plus after-glow time for each specimen after the second flame application	≤30 s	≤60 s	≤60 s
After-flame or after-glow of any specimen up to the holding clamp	No	No	No
Cotton indicator ignited by flaming particles or drops	No	No	Yes

**Table 3:** Burning test results in the horizontal and vertical directions on the samples of XPS and EPS Type 1 and 2.

		KS M ISO 9772 Horizontal Test Result			
		HF-1	HF-2	HBF	N/A
UL 94 Vertical Test Result	V-0	EPS-2-18~19	XPS-3~5, XPS-8 EPS-1-1, EPS-1-3~24, EPS-2-4, EPS-2-6~7, EPS-2-9~11, EPS-2-13~14, EPS-2-16~17, EPS-2-20~24, EPS-2-27~29, EPS-2-31~33		
	V-1				
	V-2	XPS-2, XPS-7, XPS-9 EPS-1-25, EPS-2-1~3, EPS-2-5, EPS-2-8, EPS-2-12, EPS-2-15, EPS-2-23, EPS-2-25~26, EPS-2-30, EPS-2-34			XPS-1
	N/A				XPS-6, EPS-1-2

**Table 4:** Horizontal and vertical burning test results of FR-EPS.

Scheme	KS M ISO 9772 Horizontal Test Result				UL 94 Vertical Test Result			
	Damaged Length for Each Individual Specimen [mm]	After-Flame Time Plus After-Glow Time for Each Individual Specimen [s]	Cotton Indicator Ignited by Flaming Particles or Drops	Result	Total After-Flame Time for Any Condition Set [s]	After-Flame or After-Glow of Any Specimen up to the Holding Clamp	Cotton Indicator Ignited by Flaming Particles or Drops	Result
S-1	7	0	No	HF-1	20	No	No	V-0
S-2	12	0	No	HF-1	15	No	No	V-0
S-3	13	0	No	HF-1	0	No	No	V-0
S-4	12	0	No	HF-1	0	No	No	V-0
S-5	12	0	No	HF-1	0	No	No	V-0
S-6	10	0	No	HF-1	0	No	No	V-0
S-7	9	0	No	HF-1	0	No	No	V-0
S-8	12	0	No	HF-1	0	No	No	V-0
S-9	6	0	No	HF-1	0	No	No	V-0
S-10	13	0	No	HF-1	0	No	No	V-0

**Table 5:** Results of FR-EPS samples according to the cone calorimeter method.

Specimen No.	Thermal Conductivity [W·m^−1^·K^−1^]	Peak HRR [kW∙m^−2^]	t Peak HRR [s]	TTI [s]	THR [MJ∙m^−2^]	
					at 5 min	at 10 min
SS-1	0.037	42.975	50	2	4.7	4.7
SS-2	0.038	61.129	40	3	5.9	7.9
SS-3	0.036	66.809	46	3	5.4	6.8
SS-4	0.038	54.933	50	3	5.4	6.8
SS-5	0.037	55.935	54	3	6.6	8.0
SS-6	0.038	43.231	60	2	5.4	6.9
SS-7	0.038	55.865	48	3	6.0	8.0
SS-8	0.036	46.353	48	2	4.8	4.8

**Table 6:** Cone calorimeter results on peak HRR, THR, TTI values of polystyrene composites.

Ref.	Material of Flame Retardant	Type of Composite	Peak HRR [kW∙m^−2^]	THR [MJ∙m^−2^]	TTI [s]
[35]	N/A	Pure	550	64	1
	Expanded Graphite	Compounding	130	18	3
[36]	N/A	Pure	296	42	1
	Pentaerythritol + CaCO_3_ + Melamine Cyanurate	Coating	65	11	1
[37]	N/A	Pure	738	119	63
	Expanded Graphite	Compounding	191	59	54
[38]	N/A	Pure	730	117	47
	Expanded Graphite + Aluminum hypophosphite	Compounding	163	71	23

## Abbreviations

EG	expanded graphite
EPS	expanded polystyrene
HRR	heat release rate
KS	Korean standard
PF	phenolic foam
PIR	polyisocyanurate
PUR	polyurethane
THR	total heat release rate
XPS	extruded polystyrene
